# Tobacco Smoking and Liver *Cancer* Risk: Potential Avenues for Carcinogenesis

**DOI:** 10.1155/2021/5905357

**Published:** 2021-12-10

**Authors:** Divya Jain, Priya Chaudhary, Nidhi Varshney, Khandaker Sabit Bin Razzak, Devret Verma, Tasnim Reza Khan Zahra, Pracheta Janmeda, Javad Sharifi-Rad, Sevgi Durna Daştan, Shafi Mahmud, Anca Oana Docea, Daniela Calina

**Affiliations:** ^1^Department of Bioscience and Biotechnology, University of Banasthali Vidyapith, Rajasthan, India; ^2^Department of Public Health, American International University, Dhaka, Bangladesh; ^3^Department of Biotechnology, Graphic Era (Deemed to Be University), Dehradun- 248 002 Uttarakhand, India; ^4^Department of Genetic Engineering and Biotechnology, East West University, Dhaka, Bangladesh; ^5^Facultad de Medicina, Universidad del Azuay, Cuenca, Ecuador; ^6^Department of Biology, Faculty of Science, Sivas Cumhuriyet University, 58140 Sivas, Turkey; ^7^Beekeeping Development Application and Research Center, Sivas Cumhuriyet University, 58140 Sivas, Turkey; ^8^Genetic Engineering and Biotechnology, University of Rajshahi, Rajshahi, Bangladesh; ^9^Department of Toxicology, University of Medicine and Pharmacy of Craiova, Craiova, Romania; ^10^Department of Clinical Pharmacy, University of Medicine and Pharmacy of Craiova, Craiova, Romania

## Abstract

Smoking a cigarette generates over 4000 chemicals that have a deleterious impact on each part of the human body. It produces three main severe effects on the liver organ: oncogenic, immunological, and indirect or direct toxic effects. It results in the production of cytotoxic substances, which raises fibrosis and necro-inflammation. Additionally, it also directs the production of pro-inflammatory cytokines tumour necrosis factor alfa (TNF-*α*) and interleukins (IL-1*β*, IL-6) that will be responsible for the chronic liver injury. Furthermore, it gives rise to secondary polycythemia and successively raises the turnover and mass of red cells, which might be a common factor responsible for the development of oxidative stress in the liver due to iron overload. It also produces chemicals that are having oncogenic properties and raises the risk of liver cancer especially in sufferers of chronic hepatitis C. Smoking modulates both humoral and cell-mediated responses by restricting the proliferation of lymphocytes and inducing their apoptosis and ultimately decreasing the surveillance of cancer cells. Moreover, it has been determined that heavy smoking impacts the response of hepatitis C patients to interferon (IFN) therapy through different mechanisms, which can be improved by phlebotomy. Efforts are being made in different nations in decreasing the prevalence of smoking to improve premature death and ill effects of their nation's individuals.

## 1. Introduction

Smoking in tissues that do not have direct interaction with the smoke itself, like the liver, induces different forms of unfavourable results. To defy rational reason, the popularity of smoking continues to rise, which makes many smokers recognize the harmfulness of smoking, and many of them acknowledge that they do not like it, but they remain to continue to smoke [1]. There is a behavioural mechanism that acquires the psychological and physiological addictive mood among users, which can be defined by using tobacco in any form. Nicotine, which is highly addictive and contributes to continued cigarette use, is the active ingredient in tobacco. The consumption of tobacco products is split into fuel and nonfuel. Fuel products include pipes, water pipes (hookah), small cigars, cigars, and cigarettes. Tobacco formulations and electronic cigarettes created for snuffing, dipping, and chewing are noncombustible tobacco products [2].

The strong desire to smoke is generated by nicotine from cigarettes [3]. This is the reason for the negative effects of smoking that undermines and overwhelms concerns. And also in those who try to stop smoking, resolve not to smoke. Patients complain about facial flushing episodes, arthralgia, pruritus, prickling sensation, lethargy, dizziness, throbbing headache, and the warmth of the soles and palms of the feet, which are smokers' clinical pathology. A major impact that follows the pathogenesis of liver disease was caused by heavy smoking, and response to interferon therapy among the patients with chronic hepatitis was also overlooked. Many nations are attempting to make progress by reducing cigarette consumption, but it continues to be one of the world's main reasons for poor health and early demises [4].

In light of these aspects, it is obvious that smoking as a lifestyle factor has multiple negative effects on health and that smoking cessation together with pharmacological treatments must be prioritized [5]. This article summarizes the most important side effects on the human health of smoking with a key emphasis on liver carcinogenesis to better understand the molecular mechanisms of liver toxicity and carcinogenesis associated with chronic smoking. Nonpharmacological and pharmacological therapies for the treatment of smoking are also briefly described.

## 2. Review Methodology

For this updated review, searches were conducted in specialized databases such as PubMed, Scopus, and Web of Science for all full-text articles published in English using the following MeSH terms: “humans,” “nicotine/pharmacokinetics,” “neoplasms/epidemiology,” “neoplasms/etiology,” “Smoking/metabolism,” “tobacco smoking/adverse effects,” “smoking/epidemiology,” “lifestyle,” “liver neoplasms/epidemiology.”

The study included articles on the molecular mechanisms of smoking on the liver and their correlation with carcinogenesis, ways to reduce the side effects of smoking, nonpharmacological measures, and current pharmacological therapies to combat smoking.

Abstracts, comments, and papers written in languages other than English that did not describe potentially carcinogenic mechanisms, and articles that did not contain nonpharmacological and pharmacological measures for smoke cessation were excluded from the study.

## 3. Nicotine and Other Cigarette Smoke Constituents

Smoking tobacco affects the body of human beings in myriad ways, causing lifelong illnesses and cancers to evolve. The health risks are seen not only in users but also in second-hand smoke-exposed people, and these are based on the length of smoking over the years and the time exposure to smoke from nicotine (tobacco) [6].

Various complex events are reported in response to the exposure of tobacco smoke-directed free radicals, which leads to increased DNA damage, inflammation, and oxidative stress [7, 8]. During smoking, the tar from cigarettes enters the blood, makes the blood thicker, and increases the rate of clot formation. It also raises blood pressure and heart rate, makes the working of the heart difficult, and narrows the arteries, which reduces the oxygen-rich blood circulation to organs and increases the chances of stroke [9, 10].

**Figure 1:**
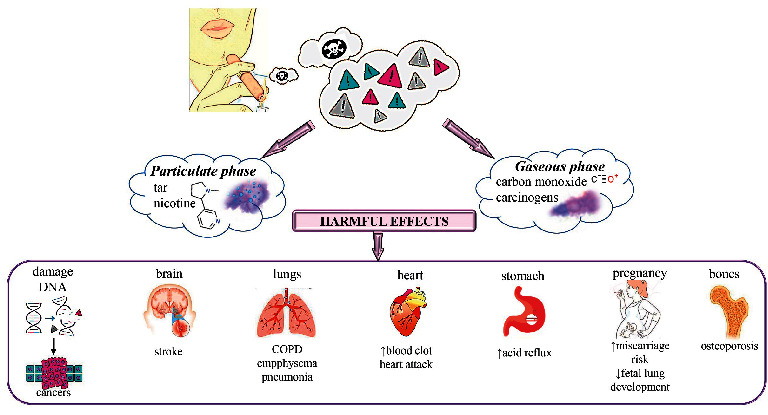
The most important harmful effects of tobacco smoking on human health.

Nicotine (C_10_H_14_N_2_) is an alkaloid tobacco plant that is responsible for its toxic nature as the main tobacco factor [11] (Figure 1). As a ganglion nicotinic cholinergic agonist, nicotine acts in the adrenal medulla, neuromuscular junction, spinal cord, cortex, and autonomic ganglia. It has pharmacological effects that are dose-based and also has both stimulant and depressant activity [11].

The use of nicotine includes a sense of pleasure and relaxation [12]. The desire to consume tobacco coincides with a low amount of blood nicotine in addicted addicts as if smoking were a way of achieving a certain degree of nicotine, reaping the rewarding sensation associated with nicotine, and preventing withdrawals. Nicotine retention in the bloodstream, if abstinence is tried, contributes to a more substantial withdrawal response. Anxiety, trouble concentrating, irritability, and extreme nicotine cravings are common symptoms of withdrawal. Withdrawal signs occur within 24 hours that can last for days, weeks, or longer. Higher nicotine levels are found in cigarettes, and their smoking suppresses the signs of nicotine withdrawal [12].

Cigarette nicotine is transported into the lungs by inhaled tar particles where a broad alveolar surface region facilitates accelerated incorporation into the circulation of the pulmonary system. Nicotine is well dispersed with a distribution volume of around 2.6 L/kg. The inactive metabolite, cotinine, mostly undergoes hepatic metabolism (80–90 percent), with most of the metabolism going to take place in the lungs and kidneys. Nicotine has a 1–4 h half-life and roughly 2–35 percent stable excretion in the urine [13].

Other cigarettes smoke constituents are present in either a gas phase or particulate phase:

1. Particulate-phase components include 4-aminobiphenyl, carbazole (tumour accelerators), indole, nicotine (ganglion stimulator and depressor), trace elements (carcinogens), catechol, cresol, phenol, polynuclear hydrocarbons, and tar [11, 14] (Figure 1)
2. gas phase contains vinyl chloride (carcinogens), hydrazine, oxides of nitrogen, nitrosamines, formaldehyde, ammonia, acrolein, acetaldehyde, hydrocyanic acid, and carbon monoxide (impairs oxygen utilization and transport) [11, 14] (Figure 1).

Smoking affects various parts of the body such as the brain, heart, lungs, stomach, skin, bones, mouth, eyes, and throat. It increases the chances of getting a stroke, which may cause brain damage. [15, 16] Smoking can also affect our central nervous system as nicotine is a mood-changing drug and addictive. Physical withdrawal of nicotine can impair cognitive functioning and can make an individual depressed, irritated, and anxious [15, 17].

It raises the risk of coronary heart diseases and strokes and cerebrovascular disease [18, 19]. Carbon monoxide and nicotine that is released from the smoke affect the normal functioning of the heart and hence increase the risk of [20] blood clots and double the risk of a heart attack [21, 22].

Lungs can be adversely affected due to smoking and can result in certain fatal diseases such as lung cancer, emphysema, pneumonia, and COPD, which is a chronic obstructive pulmonary disease in which the narrowed airways cause difficulty in breathing [23]. Smoking has effects on the throat and mouth [24]. It causes gum disease and damage to the sense of taste and raises the risk of cancer related to throat, tongue, lips, gullet, and voice box. Smokers have also a greater risk of having stomach cancer as smoking can weaken the muscle that regulates the lower end of the oesophagus and enables acid reflux in which the acid of the stomach flows backwards into the food pipe [25].

Due to smoking, the amount of oxygen to our skin is reduced, which causes early skin ageing [26, 27]. Accumulation of toxins in the body causes cellulite. It also raises the chances of developing squamous cell carcinoma (skin cancer) and fungal nail infections, and increases hair loss, balding, and greying [28].

Smoking makes bones weak and brittle and may cause osteoporosis in women [29]. It can also cause diseases related to bones such as rheumatoid arthritis [30]. It also raises the risk of cataracts and can also direct age-related macular degeneration (AMD) and type-2 diabetes mellitus [31].

Other studies have shown that smoking has accelerated the progression of chronic diseases such as kidney, heart, or pancreatic diseases. Smoking is also a prominent factor in causing kidney cancer [32].

Reproductivity and fertility can also be affected by smoking, as it decreases the count of sperm and results in testicular cancer in men. It can also cause infertility in women and increase the risk of cervical cancer. Smoking during pregnancy can cause abnormal in-utero lung development and can also cause miscarriage [33]. Nicotine is the major component that is responsible for affecting fetal lung development. Furthermore, multiple studies have shown problems such as asthma in the child of a mother who smokes during pregnancy [34].

Along with the active smoker, the people around them are also affected by second-hand smoke, which makes them susceptible to fatal diseases such as lung cancer. Children are exposed to second-hand smoke from adults, which has increased the diseases such as asthma and degeneration of pulmonary function [35].

## 4. Tobacco Smoking, Immunity, Liver, and Carcinogenesis: Connecting the Dots

### 4.1. Immune Impact of Smoking

The immune system is a diverse network of multiple components and signals [20, 36]. They help in the amplification of invader response and allow regulatory control of the overall immune reactions that contribute to the neutralization and eradication of foreign agents such as toxins or biological agents [37, 38]. This dynamic network, which can lead to immunity or autoimmune therapy or which can be blocked by a combination of biological and chemical agents, can be triggered.

In direct interaction with the natural environment, the mucosal surfaces are a large site of antigenic and harmful infection in cigarettes. Over 4,000 harmful chemicals, several carcinogens, as well as a potent immunomodulator, lipopolysaccharide, have been identified in tobacco smoke (LPS) [39]. Toxins, including endotoxins associated with cigarette smoke, can induce inflammation that leads to coronary heart disease and change humoral and cell-mediated immunity [40].

The immune system is weakened by tobacco smoke, which makes an individual susceptible to infections such as pneumonia and influenza. Many studies have shown that both adaptive and innate immunity is affected by cigarette tobacco. Smoking alters the homeostasis of the immune system, thus causing multiple diseases by exerting an abnormal effect on immune tissues and cells by modulating signalling pathways and histone changes.

**Figure 2:**
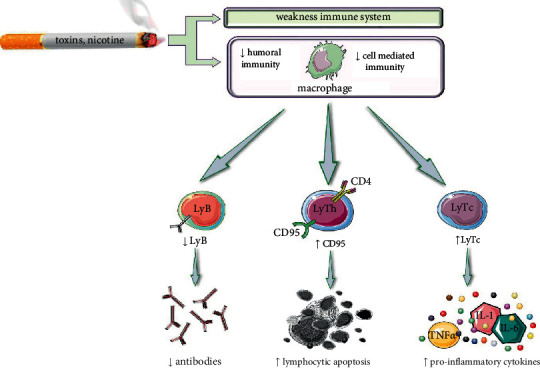
Diagram with summarized effects of tobacco smoking on the immune system. Abbreviations and symbols: ↑: increased; ↓: decreased; LyB: lymphocytes B; LyTh: lymphocytes T helper; LyTc: cytotoxic T lymphocytes; IL: interleukins, TNF-*α*: tumour necrosis factor alfa.

The impact of smoking is on both the responses, that is, humoral immune and the cell-mediated response. Nicotine hinders the differentiation and proliferation of T lymphocytes with the suppression of antibody-forming cells by inhibiting ribonucleotide reductase (RNR) and signal of antigen mediation in T-cells. Moreover, the induction of lymphocytic apoptosis by enhancing the expression of the Fas (CD95) death receptor takes place due to smoking, which is allowed to be killed by Fas ligand (FasL) which is a protein expressed on the surface of cells. There is an increase in T-cytotoxic lymphocytes (CD8+) and pro-inflammatory cytokines (TNF-*α*, IL-6, IL-1) and a decrease in CD4+ cells and impaired NK cell activity due to the effect of smoking (Figure 2).

Cessation of smoking can reverse the effects of smoking despite the adverse long-term effects and the changes, which can still be visible after a month of cessation. The effects such as increment in NK cell activity with cell-mediated and antibodies immune response as well as increased antioxidant activity decreased pro-inflammatory cytokines can be detected.

### 4.2. Impacts of Tobacco on the Liver

Smoking produces many harmful effects on many organs that have no direct interaction with smoking or smoking itself, such as the liver. The liver is an essential organ responsible for the biotransformation of drugs, narcotics, alcohol, and other harmful substances [41].

Cigarette tobacco can cause a variety of chronic liver diseases, such as nonalcoholic fatty liver disease (NAFLD) or hepatocellular carcinoma (HCC). There are also epidemiological studies that highlight smoking increases the risk of primary biliary cirrhosis (PBC) and may increase the chance of liver fibrosis in patients with chronic hepatitis B virus or hepatitis C virus and may decrease the efficacy of antiviral treatment [42].

#### 4.2.1. Tobacco Smoking and Liver Cancer: From Lifestyle to Potential Carcinogenesis

There are oncogenic potential chemicals present in smoke such as tar, vinyl chloride, nitrosamines, and hydrocarbons [43]. A major source of 4-aminobiphenyl, a hepatic carcinogen involved as a risk factor for HCC, is cigarette smoking.

**Figure 3:**
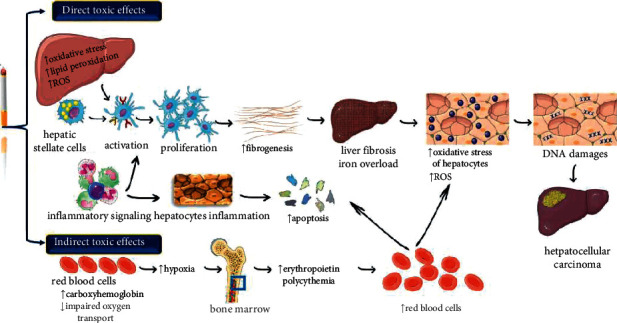
Diagram with potential molecular mechanisms by which tobacco smoking can induce liver carcinogenesis. ROS (reactive oxidative specie), ↑ (increase), ↓ (decrease).

Smoking raises HCC risk in patients with viral hepatitis. In hepatocytes that induce fibrosis, heavy smokers accumulate excess iron and promote HCC development. Hepatocarcinogenesis with HBV and HCV develops when smoking is a cofactor [44] (Figure 3). In HCC, 4-aminobiphenyl, a hepatic carcinogen, acts as a risk factor by smoking cigarettes. In HCC patients with viral hepatitis, there's an increase in risk due to smoking [44, 45].

It has been shown to have an association of smoking with liver cancer patients who are independent of HBV status, as shown in recent data from Taiwan and China [46]. Tumour suppressor gene p53, which is considered “genome guardian,” is associated with gene reduction due to tobacco smoking. The presence of tar and nicotine suppresses T-cell responses for tumour cell examination [47].

Excess of iron is present in heavy smoker's hepatocytes that is responsible for inducing fibrosis and HCC development. Smoking also is acting as a cofactor with HCV and HBV for hepatic cancer. In addition, a common feature that increases the chances of developing cancer in a heavy smoker is suppressed mood [47].

The carcinogens in tobacco smoke cause damage to cellular DNA, causing DNA disruption and gene mutations. These genetic variations cause uncontrolled cell proliferation and suppress natural processes that normally limit atypical cell growth, leading to cancer [48].

The potential mechanisms are the following:

1. Direct toxic effects: smoking may cause detrimental effects on organs that are not directly related to smoke such as the liver. Smoking raises the production of pro-inflammatory cytokines TNF-*α*, IL-6, and IL-1 that are included in the liver cell injury [44] (Figure 3). It produces substances with cytotoxic potentials; these chemicals induce direct oxidative stress related to the peroxidation of lipid, which leads to the activation of stellate cells and initiation of fibrosis. Smoking generates toxins that induce necro-inflammation and causes hepatic lesions. It also contributes to the development of HBV-related cirrhosis. Smoking may cause iron deposition, which may cause liver fibrogenesis [44].
2. Indirect toxic effects: Smoking is related to raised carboxyhaemoglobin and reduced oxygen-carrying capacity of red blood cells (RBCs), leading to tissue hypoxia. Hypoxia stimulates the generation of erythropoietin, which directs hyperplasia of the bone marrow. As a result, secondary polycythemia and increased red cell mass occur, thus contributing to the oxidative stress of hepatocytes [44] (Figure 3).

#### 4.2.2. Tobacco, Inflammation, Oxidative Stress, and Hepatocellular Carcinoma: Connecting the Dots

**Figure 4:**
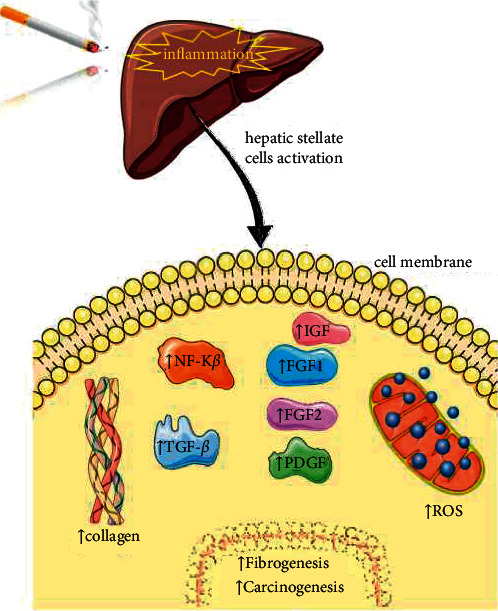
The most important molecular mechanisms and the link between inflammation, ROS, and liver carcinogenesis. Abbreviations and symbols—↑: increase; ↓: decrease;: FGF: fibroblastic growth factors; PDGF: platelet-derived growth factor; ROS: reactive oxygen species; TGF-*β*: transforming growth factor-*β*; IGF: insulin-like growth factor.

Tobacco smoking causes inflammation and chronic liver damage that activates liver stellate cells (HSCs), the key element in the initiation and progression of liver disease, a major risk factor in the development of hepatocellular carcinoma [49] (Figure 4).

### 4.3. Activated HSCs

1. Stimulate excessive collagen type I synthesis, the major constituent of the extracellular matrix. Its accumulation and damage of collagen degradation are responsible for the appearance of liver fibrosis [50].
2. Significantly increase the activity of signalling pathways mediated by kappa B nuclear factor (NF-kB) and extracellular signal-regulated kinase (ERK). NF-kB and MAP/ERK kinases stimulate the proliferation and inhibition of tumour cell apoptosis favouring carcinogenesis [51].
3. Initiate autocrine signalling mediated by transforming growth factor-*β* (TGF-*β*) and by the accumulation of *β*-catenin in the nucleus of neoplastic hepatocytes. TGF-*β* stimulates the tumour progression of neoplastic hepatocytes and induces the transformation of epithelial cells into mesenchymal cells [51, 52].
4. Stimulate growth factors such as platelet-derived growth factor (PDGF), fibroblastic growth factors (FGF1, FGF2), and insulin-like growth factor (IGF) [51].
5. Are an important source of reactive oxygen species (ROS) and pro-inflammatory cytokines IL-1*β*, IL-6, and TNF-*α* involved in hepatic fibrogenesis [51].

In chronic hepatic injury of tobacco smoking, oxidative stress is induced at the molecular level playing a pivotal role in fibrogenesis and carcinogenesis [53, 54]. Damaged liver parenchymal cells release ROS, which stimulate cell degradation and activate HSCs favouring the increase of collagen synthesis. ROS have pro-inflammatory effects and sensitize hepatocytes to apoptotic stimuli, thus being involved in fibrogenesis and liver carcinogenesis [52, 53].

#### 4.3.1. Liver Cell Damage of Tobacco among Chronic Hepatitis C Patients

The association between heavy smoking and damage to liver cells by apoptosis, excess iron deposition, and necro-inflammation in the liver has been reported. Iron overload is responsible for the deposition of iron in hepatocytes, and the induction of lipid peroxidation and oxidative stress may occur due to excess iron in the liver [55] (Figure 3).

Also, chronic HCV infections lead to damage to liver tissue. A recent study showed that in patients with chronic hepatitis C, there is a significant relationship between liver damage and cigarette smoking, especially if other factors such as alcohol consumption and age are associated [56, 57].

Smokers with chronic liver disease (hepatitis C) are reported to have a low rate of response towards the interferon (IFN) therapy. Therapeutic phlebotomy improves the rate of response within the sufferers of chronic hepatitis C to IFN therapy [58]. Furthermore, it is advised that chronic hepatitis C sufferers should quit smoking before commencing IFN treatment.

Various mechanisms have been proposed for heavy smokers in response to resistance to IFN therapy [59]:

1. The immunosuppression due to heavy smoking results in the depletion of CD4+ cells, impairment in the cytotoxic activity of NK cells, and identification of virus-contaminated cells, and induction of lymphocyte apoptosis
2. Chain smoking raises the level of iron in the hepatic organ, which provides resistance to IFN.
3. Smoking directed the production of pro-inflammatory cytokines (IL-1*β*, IL-6, and TNF-*α*) that mediates steatosis and necro-inflammation.
4. Tobacco smoking modulates the IFN-*α*-directed cell action and signalling through the unresponsiveness of T-cells. Through these mechanisms, it has been determined that smoking that is an underrated risk factor for hepatic ailments needs further investigation for the solution of this issue.

## 5. Strategies on Smoking Cessation

It can be divided into three main approaches: methods for public health, and therapeutic and alternative approaches.

### 5.1. Nonpharmacological Interventions for Smoking Cessation

Smoking prevention clinical approaches include telephone counselling, self-help programs, cognitive-behavioural approaches such as client and group counselling, interventions by service providers, and fitness programs. Alternative treatments include acupuncture, aversive treatment, and hypnosis. Multimedia interventions, workplace interventions, and community-level interventions changes in public policies provide approaches to public health [60].

Cigarettes, cigarillos, cigars, and pipes are the most common type of smoked products. Globally in some areas, “smokeless tobacco” is also popular. Usually, it involves preparations for sniffing into the nose, chewing, or placing in between gums and cheeks as a wad. Smokeless tobacco and smoking features are similar to each other and can cause major health risks [61].

Quit attempt for smoking usually involves an intention from a given point of time to not to smoke any more cigarettes, by following the self-conscious struggle of smoking desire resulting in abstinence period. If a person smokes a few numbers cigarettes on any occasion but then restarts abstinence, this is known as “lapse.” If the person continues smoking daily, then the person is having “relapsed.” The 4 weeks of abstinence is defined as “short-term abstinence,” and abstinence for at least 6 months to 12 months is referred to as “long-term abstinence.” It is very important to know about the time duration for the period of abstinence before deciding and using this term when someone quit smoking as there is no fixed criterion [61].

### 5.2. Current Pharmacological Treatment of Cigarette Smoking

In addition to nonpharmacological interventions for smoking reduction, pharmacologic treatment should be included [6]. Seven FDA-approved smoking cessation drugs are available: nicotine replacement therapy, bupropion sustained release (SR), varenicline, nicotine gum, nicotine lozenge, transdermal nicotine patch, and nicotine inhaler. According to the United States Public Health Service guidelines, these drugs should be called first-line treatment [62].

#### 5.2.1. Nicotine Replacement Therapy

Nicotine replacement therapy (NRT) medicines reduce symptoms of craving and withdrawal by progressively eliminating the lack of nicotine intake. Five products with nicotine replacement treatment (NRT) are approved by the US. Tobacco dependency treatment in Food and Drug Administration of the transdermal patch with nicotine, nicotine inhaler, nicotine nasal spray, nicotine lozenge, and nicotine gum. The prescribed medications in the United States are the nicotine inhaler and nasal spray, whereas the patch, lozenge, and nicotine gum are sold as over-the-counter products [63].

NRTs function by partly removing nicotine obtained from cigarette use to decrease the severity and duration of withdrawal symptoms. Generally, NRT is well tolerated with minimal side effects. In clinical trials, vomiting, nausea, headache, and other stomach complaints were the three most frequently recorded adverse effects of NRT. Their side effects are usually unique to the formulation, depending on the distribution method used (Hays, 2010). With mild adverse effects, NRT is usually well tolerated. In observational reports, headache, nausea and vomiting, and other stomach complaints were the three most frequently recorded side effects of NRT [63].

1. Nicotine patch: Transdermal patches are intended to slowly and gradually release nicotine. Nicotine transfer takes place as soon as the patch is applied until a constant state is reached with the presence of nicotine in the patch, skin “reservoir,” and circulation. It increases the rate of cessation by around 1.5 to 2 times compared to placebo when used alone [64].
2. Nasal sprays: Among all nicotine replacement products, the quickest distribution and maximum nicotine rate, and the long-term withdrawal are around doubled. The 0.5 mg of nicotine is administered by simple spray, and a single dose (1 mg) is sprayed into each nostril. Absorption takes place via the nasal mucosa, and maximal concentrations of plasma nicotine are achieved within 10–15 minutes. The 1–2 doses per hour should be prescribed, and the dosage should then be titrated to a limit of 40 mg a day per individual requirement [64].
3. Nicotine inhaler: It was launched in 1998 and is more like a “puffer” than an inhaler, made of a plastic tube containing a cartridge of nicotine. The cartridge contains 4 mg of nicotine vapour from a porous plug that is then absorbed from the mouth. The continuous abstinence rates of nicotine inhalers have been seen twice [64].
4. Nicotine sublingual tablets and lozenges: Microtablets that sublingually release nicotine and sugar-free nicotine lozenges are more recent oral forms of nicotine therapy. In contrast to nicotine gum, lower nicotine is offered in micropills, so a daily dose of 80 mg per day must be taken more regularly. On the other side, lozenges contain 25% and 27% more than nicotine gums, respectively. There should be at least 7–8 lozenges per day with a limit of 25 lozenges per day [64].

#### 5.2.2. Nicotine Vaccine

The development of a nicotine-specific vaccine is a novel method to deal with smoking cessation. The binding of nicotine to a sufficient antigenic protein induces the production of antibodies (Nic-IgG) of high nicotine affinity and specificity. Nicotine is sequestered in the blood by these receptors, thereby preventing its entry into the brain. It can be given on 2–4 occasions with results that last for many months [65].

#### 5.2.3. Bupropion Sustained Release (SR)

Through its dopaminergic activity, bupropion (Zyban) greatly decreases withdrawal symptoms and should also be used to stop smoking. It can be used in abstinent smokers to avoid recurrence and minimize weight gain. Bupropion is the first non-nicotine agent and is effective in treating tobacco dependence [66]. Bupropion sustained release is FDA-approved for the prevention of smoking and is considered by the US. Public Health Service as a first-line treatment. The most common side effects of bupropion are insomnia, which occurs in around 30–40 percent of patients, and dry mouth, which occurs in 10 percent of patients. The suggested mechanisms of action of the continuous release (SR) of bupropion involve the blockade of neuronal reuptake dopamine and norepinephrine [66].

#### 5.2.4. Varenicline

The newest medicine on the market, Champix (Pfizer), has been developed particularly for smokers [67]. This is a first-line smoking reduction agent. Varenicline is a partial agonist specific to subtype alfa-4*β*2 of the neuronal nicotinic acetylcholine receptor. It binds to and induces partial stimulation of the nicotinic receptor as a partial agonist, thus reducing nicotine withdrawal symptoms [68]. Varenicline is normally well tolerated, with nausea, insomnia, and headache as the most frequent adverse events [69]. Varenicline operates by specifically binding to alfa-4*β*2 neuronal acetylcholine nicotine receptors, resulting in agonist activity, thus preventing nicotine from binding to alfa-4*β*2 receptors at the same time [67, 69].

#### 5.2.5. Clonidine

Clonidine is the second-line medicinal drug used to avoid smoking. While second-line drugs have been proven to be effective in managing nicotine dependency, their use is limited largely because of a less desirable side effect profile compared to first-line drugs [70]. Clonidine, an alpha-2-adrenoceptor antagonist used in the removal of opiates and alcohol, has also demonstrated that certain signs of nicotine withdrawal have been reduced [71]. As a second-line drug, clonidine can be used where primary treatments are shown to be unsuccessful. It is only approved by FDA for hypertension, but it is effective to stop smoking too. It is an alfa-2-adrenergic agonist whose effect on smoking is assumed to be based on its ability to overcome nicotine withdrawal characteristics of the CNS, including craving and anxiety. It is limited by its profile of adverse effects, including extreme drowsiness, postural hypotension, tiredness, and dry mouth [71].

### 5.3. Complementary and Alternative Medicine

For the patients who have failed to reach monotherapy cessation, a combination of complementary therapy with pharmacologic agents is also used. Very little research has been verified that complementary and alternative medication (CAM) for tobacco withdrawal, including meditation, hypnosis, medicinal supplements, acupuncture, calming, and massage therapy, has been attempted and is effective [72, 73].

However, the use of therapy for complementary and alternative medicine and a higher level of education have been substantially related [74]. As a promising alternative therapy for the treatment and avoidance of addictive habits, they are done by yoga and mindfulness meditation. The hypothetical models indicate that the self-awareness, perceptions, and skills adapted to yoga and mindfulness practice may target several behavioural, neural, physiological, and psychological factors that could be linked to relapse due to addiction. Other considerations such as adverse effects, data, cost, and route of delivery of medications should also be addressed by clinicians. The purpose of therapy should be to prescribe an affordable agent with demonstrated effectiveness and a strong profile of tolerability. It is also desirable to choose a medication formulation that helps patients achieve commitment to treatment [75, 76].

## 6. Overall Conclusions and Perspectives

The most prevalent mode of tobacco usage remains cigarette smoking. There are several adverse health effects associated with cigarette smoking; ongoing attempts to decrease the incidence of cigarette smoking are thus crucial. The importance of smoke reduction and smoking prevention campaigns targeting young people is demonstrated by current trends in tobacco smoking. Smoking prevention advocacy will be a good public health tool to reduce the environmental sensitivity of nonsmokers to environmental cigarette smoke. Both behavioural and pharmacological approaches should be used in managing nicotine dependence.

Researchers have recognized many chemicals of tobacco smoke as liver carcinogens. However, it is not clear up yet whether DNA adducts are enough to cause tumorigenesis or the initiation of DNA adducts in the liver organism the initiator of carcinogenesis. Consumption of tobacco has been also related to a raised risk of liver cancer with a slow rate of response to INF-*α* therapy in the sufferers of chronic hepatitis C. Different mechanisms are studied in response to resistance to IFN-*α* therapy in smoking individuals. But the exact mechanism responsible for smoking-related immunopathology, carcinogenesis, and resistance is still unclear and needs further exploration.

The health impact of smoking and the benefits of stopping are evident. The deleterious effects on oral and systemic health steadily diminish with time. The risk of repeated heart problems and premature mortality following smoking cessation is significantly diminished. The heartbeat and blood pressure drop at a regular rate. The risk of mouth, nose, oesophagus, bladder, kidney, and pancreas cancer is declining. Short-term effects include a reduction in poor breathing (RDH., 2012; Bhatnagar D and Jain DC., 2011).

Smokers who quit smoking before the age of 35 years have around a similar life expectancy as nonsmoker. After 35 years of age, it recovers only 2 to 3 months or 4 to 6 hours/day of life expectancy. Quitting smoking at any age is advantageous with the comparison of smoking. In some ailments, the chances of risk get reversed, whereas in others, the risk is almost frozen at the point when smoking stopped.

In conclusion, tobacco smoking and the mechanisms involved in the initiation and progression of hepatocellular carcinoma are not strictly delimited. Direct effects on the liver, indirect effects on the body, the role of inflammation, and ROS generation contribute directly.

To the formation and activation of the pathways involved in fibrogenesis and carcinogenesis. The clinical perspectives of this article derive from a good understanding of the pathophysiological mechanisms by which activated HSCs have a role in carcinogenesis, and thus, new therapeutic targets can be developed in the future to aim at their inhibition.

## Data Availability

The data supporting this systematic review were taken from previously reported studies and datasets, which have been cited. The processed data are available from the corresponding author upon request.
